# Supplementary material for: Examining the Predictors of Mental Ill Health in Esport Competitors
Source: Healthcare (Basel). 2022 Mar 26;10(4):626. doi: 10.3390/healthcare10040626 (PMC9027719; doi:10.3390/healthcare10040626)
Supplement: Supplementary file 1 [file healthcare-10-00626-s001.zip › healthcare-1572909-supplementary.pdf]

Supplementary material 1 (S1): Information about calculating stressor categories

All stressors were subjected to factor reduction by conducting principal component analysis (see Table below), a data-led technique is used to maximize the variability and reduce the dimensionality of a dataset (e.g., Amieva et al., 2003; Bennett et al., 2005). A four-fold approach was followed as a method for appropriately interpreting PCA output: parallel analysis (Horn, 1965), Kaiser criterion (> 1; Kaiser, 1960), inspection of Cattell's scree plots (Cattel, 1952) and the interpretability (Fabrigar et al., 1999). Orthogonal Varimax rotation was applied to the component matrix (Kaiser, 1958). Varimax rotation seeks to increase the variances of the factor loadings (i.e., factor loadings above 0.4 were considered significant) and the greater scores were maintained when cross-loading between components were present (Howard, 2016). Seven factors were extracted from the PCA with eigenvalues greater than one (see figure 1), latent variables were produced for each individual by regressing task scores onto the rotated component matrix.

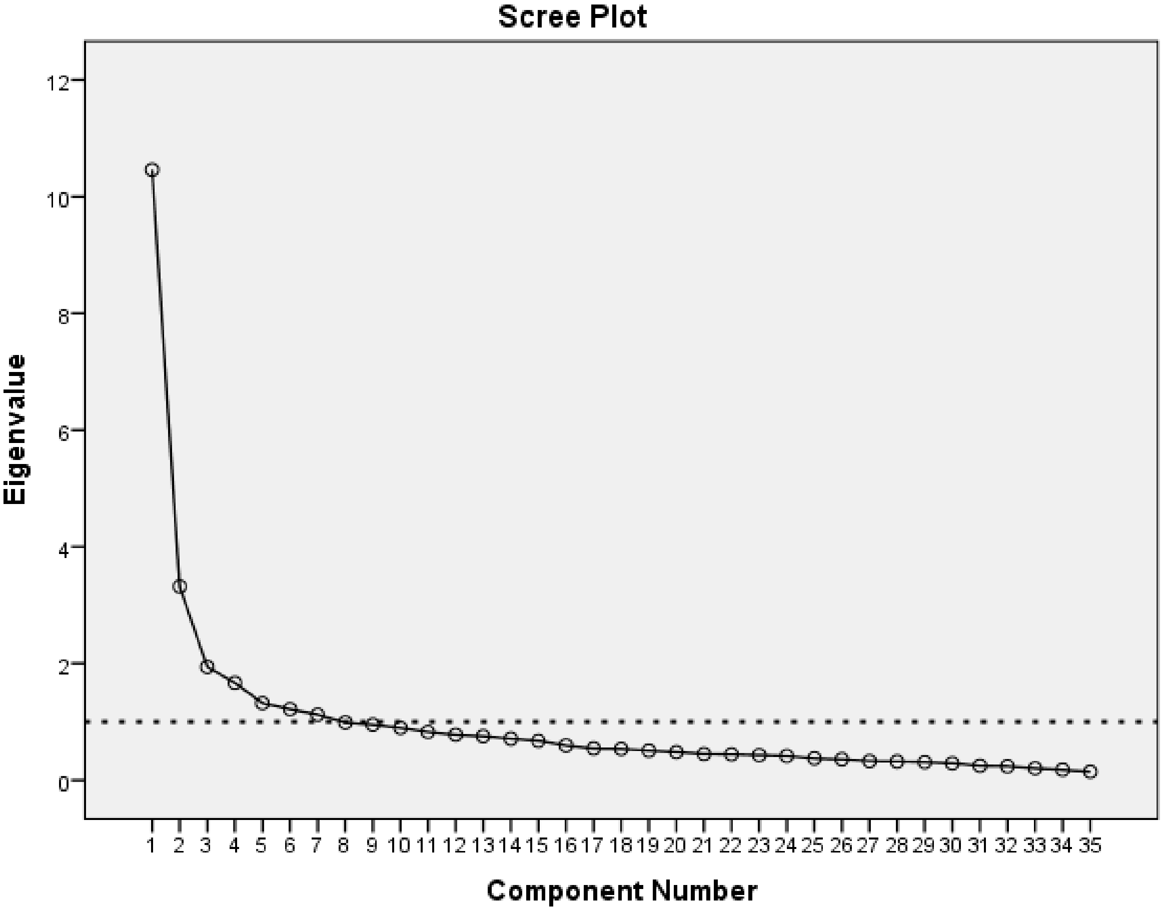

Figure S1. *Scree plot produced through PCA, demonstrating 7-component solutions (eigenvalue >1), used for calculating individual latent stressor variables. Dotted line highlights eigenvalue cut-off (Kaiser, 1960).*

Table S1. *Rotated component loadings on all stressors.*

**Stressors**	***Component***						
	1	***2***	***3***	***4***	***5***	***6***	***7***
Frustration from lack of in-game communication	0.751						
Teammates not listening to IGL (In Game Leader)	0.801						
Teammates not following instructions	0.802						
Teammates valuing individual statistics over team success	0.619						
Aggressive communication from IGL					0.701		
Need to contribute to the team		0.531					
Playing in the deciding round							0.735
Being on a run of bad form		0.521					
Other players getting emotional					0.661		
Lack of support from teammates	0.484						
Lack of clarity on game plan	0.401						
Knowing you're the last player on your team in the round							0.765
Negative/critical communication from IGL					0.691		
Worry that teammates will be affected by 'trash-talk'					0.308		
Teammates not putting in the practice time			0.814				
Losing a round you expected to win				0.7			
When it gets close in a game							0.53
Losing a map you expected to win				0.75			
Negative comments about IGL decisions						0.554	
Controlling communication from IGL					0.613		
Not knowing how break the other teams momentum				0.49			
Teammates not focussed in game	0.534						
Worries about your skill level		0.735					
Fear of failure		0.665					
Teammates not taking practice seriously			0.777				
Certain maps you're not strong on				0.449			

Having to have good statistics	0.688		
Worry about criticism and being dropped	0.685		
Uncertainty over my own teams decision-making			0.492
Teammates' lack of practice hours		0.803	
Organising practice and competition around other 'life' aspects		0.384	
Trash talk from opposition when your team are losing/on-tilt			0.741
Concern about your teammates' emotional stability			0.637

Note. Applied rotation method is varimax.

Organization and Labelling of Stressor Categories

PCA1) Teammate interactions – Problems with interactions with teammates

1-Frustration from lack of in-game communication
2-Teammates not listening to IGL
3-Teammates not following instructions
4-Others valuing individual stats over team success
10-Lack of support from teammates
11-Lack of clarity on game plan
24-Teammates not focussed in game

PCA2) Personal concerns – concerns about your own individual contribution

6- Need to contribute to the team
8- Being on a run of bad form
25- Worries about their skill level
26-Fear of failure
29- Having to have good statistics (e.g., adapted from 'having to have a good average')
30-Worry about criticism and being dropped

PCA3) Teammate concerns – concerns about teammate contributions
3 - concern/lack of confidence in teammates
15- Players not putting in the practice time
27- Others not taking practice seriously
32- Teammates' lack of practice hours
33-Organising practice and competition around other 'life' aspects

PCA4) Game-specific worry – worry about aspects about the game.
18-Losing a round you expected to win
20-Losing a map you expected to win
23- Not knowing how break the other teams momentum
28- Certain maps you're not strong on

PCA5) IGL-specific interactions – problematic interactions with the IGL
5- Aggressive communication from IGL
9- Other players getting emotional
13- Negative/critical communication from IGL
22- Controlling communication from IGL

PCA6) Game-specific uncertainty -
14- Worry that teammates will be affected by 'trash-talk'
21- Negative comments about IGL decisions (teammates)
31- Uncertainty over (my own teams') decision-making
33- Organising practice and competition around other 'life' aspects
34-Trash talk from opposition when your team are losing/on-tilt
35-Concern about (your teammates) emotional stability

PCA7) In-game pressure – pressure that occurs within the game situation
12- Knowing you're the last player on your team in the round
7- Playing in the deciding round
19- When it gets close in a game

Supplementary material S2: Table S2. Descriptive statistics of outcome variables

Variable	*M*	*SD*
Social phobia anxiety - fear	14.92	6.69
Social phobia anxiety - avoidance	7.65	3.79
Social phobia anxiety - physiological symptoms	11.51	5.28
Burnout - reduced sense of accomplishment	14.06	2.79
Burnout - exhaustion	9.54	4.51
Burnout - sport devaluation	11.92	4.83
Sleep - subjective sleep quality	2.21	0.75
Sleep - latency	1.87	1.14
Sleep - duration	0.67	0.88
Sleep - efficiency	0.60	0.97
Sleep - disturbance	1.08	1.11
Sleep - medication	0.36	0.77
Sleep - daytime dysfunction	1.59	0.76
Distress screener	7.27	2.47
Severe depressive symptoms (PHQ9)	41.69	17.61
General anxiety and depression (GHQ12)	26.49	5.16

Note. M = Mean; *SD* = Standard deviation
